# Supplementary material for: Reasons for declining to participate in a trial of online cognitive behavioural therapy following orthopaedic trauma: A mixed methods study
Source: PLoS One. 2025 Jan 16;20(1):e0317485. doi: 10.1371/journal.pone.0317485 (PMC11737714; doi:10.1371/journal.pone.0317485)
Supplement: Attachment — Submitted filename: Review Comments.docx [file pone.0317485.s001.docx]

Sheila Sprague et al. explored the reasons for refusal of the COPE trial and population characteristics of fracture patients through questionnaires and telephone calls to provide information about evidence of psychotherapy and orthopaedic trauma recovery for the subsequent patient consent process for the COPE trial to facilitate patient registration. The overall analysis of the study was monotonous and the readability was more general. There are also some issues with the study that require attention:

1. the study was relatively simple, with a small sample size included and insufficient evidence;

2. single-center study, can be carried out jointly with multi-center study, and at the same time to solve the problem of small samples;

3. whether this questionnaire involves whether it is perfect? Whether the real situation is fully reflected through the questionnaire? Whether to take into account the impact of other factors: such as the patient's financial burden, the family's schedule, whether to accept this online way, the patient himself or herself has no influence by family members and the ability to complete alone, and so on.
