# Supplementary material for: Reasons for declining to participate in a trial of online cognitive behavioural therapy following orthopaedic trauma: A mixed methods study
Source: PLoS One. 2025 Jan 16;20(1):e0317485. doi: 10.1371/journal.pone.0317485 (PMC11737714; doi:10.1371/journal.pone.0317485)
Supplement: Attachment — Submitted filename: Response to Reviewers.docx [file pone.0317485.s002.docx]

September 3, 2024

**RE: Reasons for Declining to Participate in a Trial of Online Cognitive Behavioural Therapy Following Orthopaedic Trauma: A Mixed Methods Study**

Dear Editor,

Thank you for considering our manuscript entitled, “Reasons for Declining to Participate in a Trial of Online Cognitive Behavioural Therapy Following Orthopaedic Trauma: A Mixed Methods Study” for publication in PLOS ONE.

Please find attached both clean and tracked versions of our revised manuscript. We have responded to the journal and reviewer comments below.

1. Please ensure that your manuscript meets PLOS ONE's style requirements, including those for file naming.

**Response:** We have reviewed our file names and formatting and have confirmed that they are in line with PLOS ONE’s requirements. Please specify if anything has been missed.

2. We note that you have indicated that there are restrictions to data sharing for this study. PLOS only allows data to be available upon request if there are legal or ethical restrictions on sharing data publicly.

**Response:** Our ethics board and institutional policies require data to be kept confidential unless a request for data sharing is submitted and approved. Anyone interested in viewing the study data can send a request to corresponding author via email.

3. One of the noted authors is a group or consortium [COPE Investigators]. In addition to naming the author group, please list the individual authors and affiliations within this group in the acknowledgments section of your manuscript. Please also indicate clearly a lead author for this group along with a contact email address.

**Response:** Please find the individual authors and affiliations of the COPE Investigators group listed under Acknowledgments. Lead authors are Dr. Sheila Sprague ([sprags@mcmaster.ca](mailto:sprags@mcmaster.ca)) and Dr. Jason Busse ([bussejw@mcmaster.ca](mailto:bussejw@mcmaster.ca)). This information is also provided on the title page.

4. Consider providing more details on the COPE trial intervention in the methods section to give readers better context.

**Response:** We have added more details on the CBT program as requested.

5. The discussion could be strengthened by further exploring potential strategies to address the identified barriers to participation, particularly around mental health stigma and time commitment concerns.

**Response:** We have expanded our discussion on these topics as requested.

Please do not hesitate to contact me if you have any questions or concerns. Thank you for your consideration and we look forward to further communication with you.

Sincerely,

Sheila Sprague, PhD	Jason W. Busse, DC, PhD

293 Wellington St. N, Suite 110	1280 Main St. West, HSC-2V9

Hamilton, ON L8L 8E7	Hamilton, ON L8S 4K1

Email: [sprags@mcmaster.ca](mailto:sprags@mcmaster.ca)	Email: [bussejw@mcmaster.ca](mailto:bussejw@mcmaster.ca)

Tel: 905-929-7846	Tel: 905-525-9140 (x21731)
