# Supplementary material for: Reasons for declining to participate in a trial of online cognitive behavioural therapy following orthopaedic trauma: A mixed methods study
Source: PLoS One. 2025 Jan 16;20(1):e0317485. doi: 10.1371/journal.pone.0317485 (PMC11737714; doi:10.1371/journal.pone.0317485)
Supplement: Attachment — Submitted filename: Response to Reviewers (2).docx [file pone.0317485.s003.docx]

November 30, 2024

**RE: Reasons for Declining to Participate in a Trial of Online Cognitive Behavioural Therapy Following Orthopaedic Trauma: A Mixed Methods Study**

Dear Editor,

Thank you for reviewing our manuscript entitled, “Reasons for Declining to Participate in a Trial of Online Cognitive Behavioural Therapy Following Orthopaedic Trauma: A Mixed Methods Study” for consideration for publication in PLOS ONE.

Please find attached both clean and tracked versions of our revised manuscript. We have responded to the journal and reviewer comments below.

1. **Please review your reference list to ensure that it is complete and correct.**

Response: We have reviewed the reference list and added corrections for two papers (references 4 and 19) that have been published recently. None of the corrections change the utility of the cited research, so we have simply added each correction alongside the original reference.

2. **Line 92, recommend changing "will complete" to complete in past tense.**

Response: We have made this change accordingly at line 92.

3. **Line 149-151: Did any participants review their transcriptions? If so, did they agree that responses were accurately captured?**

Response: The option to read and revise the transcribed interview was offered to all participants. Five participants opted to receive copies of their interview transcriptions to review. None of the five requested changes to the transcript. This information has been clarified at lines 149-152.

4. **Line 161-162: Can you describe more specifically how you utilized Braun and Clarke's thematic analysis for your qualitative analysis? / Consider describing in more detail how applied Braun and Clarke's methods for thematic analysis.**

Response: Thank you for this revision – we have added more detail about the theming process at lines 162-164.

5. **Line 165-166: How did you strive to achieve descriptive and interpretive validity?**

Response: Thank you for this question. We have added additional detail about the separate and parallel theming process that provided increased validity to our results at line 168-169.

6. **Line 169-171: Can you describe the coding process in more detail?**

Response: Thank you for this question. Through responding to the previous 2 questions, we have added more detail about the coding process in lines 161-169.

7. **Consider including the following (for COREQ Domain 1: Research team and reflexivity): Interviewer characteristics: Bias, assumptions etc.**

Response: Thank you for this question. We have added some information about the education level of the interviewer to lines 136-140. This now represents all the information we collected about the interviewer at the time of interview.

8. **Consider including a description of the coding tree.**

Response: We have created a coding tree and added it as Figure 2, attached with this resubmission.

9. **Was the semi-structured interview guide piloted? If yes, with whom?**

Response: Thank you for this question. The interview was piloted with members of the research team who regularly work with fracture patients.

Please do not hesitate to contact me if you have any questions or concerns. Thank you for your consideration and we look forward to further communication with you.

Sincerely,

Sheila Sprague, PhD	Jason W. Busse, DC, PhD

293 Wellington St. N, Suite 110	1280 Main St. West, HSC-2V9

Hamilton, ON L8L 8E7	Hamilton, ON L8S 4K1

Email: [sprags@mcmaster.ca](mailto:sprags@mcmaster.ca)	Email: [bussejw@mcmaster.ca](mailto:bussejw@mcmaster.ca)

Tel: 905-929-7846	Tel: 905-525-9140 (x21731)
